# Role of microRNAs in type 2 diseases and allergen-specific immunotherapy

**DOI:** 10.3389/falgy.2022.993937

**Published:** 2022-09-12

**Authors:** Constanze A. Jakwerth, Hannah Kitzberger, Dimitrii Pogorelov, Annika Müller, Simon Blank, Carsten B. Schmidt-Weber, Ulrich M. Zissler

**Affiliations:** Center of Allergy / Environment (ZAUM), Technical University of Munich, School of Medicine and Helmholtz Center Munich, German Research Center for Environmental Health, Member of the German Center for Lung Research (DZL), Member of the Immunology and Inflammation Initiative of the Helmholtz Association, Munich, Germany

**Keywords:** microRNA, airway epithelial cells (AECs), type 2 inflammation, immune crosstalk, allergen specific immunotherapy (ASIT), venom immunotherapy (VIT), induced sputum, nasal secretions

## Abstract

MicroRNAs (miRs) have gained scientific attention due to their importance in the pathophysiology of allergic diseases as well as their potential as biomarkers in allergen-specific treatment options. Their function as post-transcriptional regulators, controlling various cellular processes, is of high importance since any single miR can target multiple mRNAs, often within the same signalling pathway. MiRs can alter dysregulated expression of certain cellular responses and contribute to or cause, but in some cases prevent or repress, the development of various diseases. In this review article, we describe current research on the role of specific miRs in regulating immune responses in epithelial cells and specialized immune cells in response to various stimuli, in allergic diseases, and regulation in the therapeutic approach of allergen-specific immunotherapy (AIT). Despite the fact that AIT has been used successfully as a causative treatment option since more than a century, very little is known about the mechanisms of regulation and its connections with microRNAs. In order to fill this gap, this review aims to provide an overview of the current knowledge.

## Introduction

As upper and lower airways are considered a morphologic and functional unit, not only do allergic rhinitis and asthma share mechanisms of allergic inflammation (1, 2). Also transition of allergic rhinitis into asthma can represent a steady continuum depending on endotype and is therefore of relevance with respect to allergic pathomechanisms (3, 4). Allergic diseases are characterized by an uncontrolled immune reaction towards harmless environmental antigens to which the body is exposed either *via* airways, as seen in allergic rhinitis and allergic asthma (5). Type 2-driven diseases are characterized by the infiltration of inflammatory cells into the lung, elevated IgE serum levels, mucus hypersecretion, bronchial hyperresponsiveness (BHR), airway obstruction, and chronic inflammation (6, 7). Type 2 cytokines including interleukin-4 (IL-4), IL-5, IL-13, IL-24, and IL-33 mediate allergic inflammation and airway eosinophil infiltration, which affect the function of airway wall epithelial and smooth muscle cells (1, 4, 8). These mediators can be detected in different biomatrices, which can be assessed more or less invasively (8). A functional connection between miR expression and the pathogenesis of asthma was reported for a high number of genes (9). Importantly, mRNA transcripts of multiple genes can be targeted by a single miR (9), which enables miRs to act broadly on controlling cell function.

98% of the human genome consists of non-coding DNA sequences (10). Non-coding RNAs (ncRNAs) have various functions such as gene regulation and can be divided into different groups. Long non-coding RNAs (lncRNA) are defined by being composed of more than 200 nucleotides and not being translated into protein. Their function is often not clear, because it is a big and heterogenous group. One process lncRNAs are involved in is gene regulation on the epigenetic level *via* chromatin remodelling (11–13). Small nucleolar RNAs (snoRNAs) are responsible for the fine-tuning of the ribosome and spliceosome function. They do this mostly by post-transcriptional regulation of ribosomal RNA (rRNA) and small nuclear RNA (snRNA) (14, 15). Another group are the circular RNAs (ciRNAs), which are single-stranded, covalently closed RNA molecules. They were first discovered in 1976 and thought to be viroids, pathogens of certain plants (16). The first detection in humans was in the human cancer cell line HeLa by electron microscopy in 1979 (17). The most prominent and best-investigated group are miRs, which are about 22 nucleotides long and function as post-transcriptional suppressors of gene expression by binding to a complementary mRNA sequence (18). miRs are transcribed mostly by RNA polymerase II (19), only a few by RNA polymerase III (20). They carry a 5′-cap and are polyadenylated at the 3′ tail (21). miRs are processed by RNAase II enzymes DROSHA and DICER to result in their mature form (22, 23). Its precursors are located most frequently in intergenic regions and introns of protein-coding genes (24). It is also known that one miR can have multiple mRNAs as its target (25).

miRs have been associated with different disease phenotypes. The role of miRs in human diseases has primarily been studied in cancer, showing that defects in their processing can lead to huge damage. This highlights the role of the let-7 miR family, which targets oncogenes and is reduced in cancer cells (26). This reduced post-transcriptional suppression leads to increased expression of oncogenes and thus supports tumour growth (27–29). However, the role of non-coding RNAs in allergic diseases and in central pathways of allergy pathophysiology remains largely unknown.

## Non-coding RNAs in allergic airways

Allergic diseases are caused by an excessive response to pathogens, allergens, or toxins. Allergic rhinitis, allergic asthma, and atopic dermatitis are the most common allergic diseases (30). Asthma is a very heterogenous chronic airway disease characterized by airway hyperreactivity, smooth muscle hyperresponsiveness and inflammation (31). It is characterized by a type 2 immune response that includes prototypic type 2 cytokines and elevated levels of eosinophils and IgE. miRs can contribute to the development of asthma by regulating inflammation, cell migration, and proliferation, which was shown in animal models and isolated human cells (8, 32–34). In allergic diseases characterized by a cytokine milieu dominated by type 2 mediators such as IL-4 and IL-13, epithelial cells may express miRs (Table 1), which may also be associated with a decline in lung function parameters (4). Recently, it was shown that small extracellular vesicles (sEV)-associated epithelial miRNAs are involved in regulating the immune responses by underlying dendritic cells (DCs) (35). Decreased levels of miR-92b, miR-210, and miR-34a in epithelial-derived sEVs upon asthma development, would allow DCs to polarize Th2 cells, perpetuating the asthmatic phenotype in the lung microenvironment (35). Furthermore, an association of certain miRs with airway obstruction measurements as well as with the gene expression amplitude of their predicted target genes was found. The latter are relevant to type 2 airway polarization in children and therefore suggest a role for miRs in the development of asthma. In addition, airway remodeling and loss of epithelial integrity in asthma affect airway obstruction and hyperreactivity, which may be regulated by epithelial small extracellular vesicles containing specific miRs.

**Table 1:** Micro-RNAs in type 2-driven diseases.

miRNA	Function in pathogenesis	Predicted Targets	References
let-7a	Regulates IL-13 expression	IL-13	(39)
let-7d	Decreases expression of IL-13, IL-6 and TLR4	IL-13	(58)
miR-19	promotes T_H_2 cell cytokine production through direct targeting of the signaling inhibitors *PTEN*, *SOCS1*, and *TNFAIP3 (A20)*	PTEN, SOCS1, TNFAIP3	(47)
miR-21	upregulated in allergic airway inflammation; correlated with disease risk, severity, and inflammation of AR	IL-12p35	(110, 111)
	Upregulated by IL-4		(112)
miR-34	Down-regulated in asthma		(38)
miR-34/449	Repressed IL-13	NOTCH1	(48)
miR-126	Positively correlated with the severity of the asthma; increased expression of GATA3 in T cells; associated with increased levels of IL-4 and Th17 cells	IL-4, GATA3	(113)
miR-133a	Alleviates airway remodeling in asthma through PI3K/AKT/mTOR signaling pathway IGF1R	IGF1R	(52)
miR-139	Activates the JAK3/STAT5 signaling pathway, associated with increased levels of TNF-α, IL-6, IL-8 and IL-1β	JAK3/STAT5	(114)
miR-141	Interferes with IL-13; increased goblet cell proportions, MUC5AC expression and increased secreted mucus	IL-13	(115)
miR-142-3p	Associated with aberrant WNT signaling during airway remodeling in asthma	WNT	(68)
miR-143	Increased expression of FoxP3, stimulated proliferation of CD25+ CD4 + lymphocytes, downregulated IL-13.	IL-13, FoxP3	(116)
miR-145	Promoted type 2 inflammation; induced upon allergen exposure	HMGB2, OCT4, KLF4, MUC1, JAM-A, FSCN1, IRS1	(117)
miR-155	High miR-155 levels were strongly associated with high IFN-γ production, increased airway Th1 cytokine polarization (IFN-γ/IL-4 ratios) and increased pro-inflammatory responses.	SHIP1	(118)
miR-182	Differentiation of Th17 cells	IL6ST, IL31RA, FoxO1, FoxO3	(55)
	Regulates proliferation of T cells and Treg cells function		(12, 107)
miR-186	Regulation of PTEN	IL1R1, IL13RA1, PTEN, HMGB1	(119)
miR-187	Downregulated in allergic rhinitis.	RUNX2, TEAD1, DMRT3, E2F2, PRDM1	(120)
	Upregulated in asthma		(89)
miR-190	Upregulated in allergic rhinitis	Not defined	(121)
miR-191	Correlated with FEV1% pred., eosinophil and neutrophil counts in blood	ADAM9, MAPK9, NOTCH2	(122)
miR-192-5p	Attenuated airway remodeling and autophagy in asthma by targeting MMP-16 and ATG7	CXCL2, CXCR5,	(54)
miR-204	Regulates bronchial smooth muscles cells proliferation	IL7R	(105)
miR-221	Correlated with airway eosinophilia in asthma; increased CCL-24, CCL-26, and POSTN in airway epithelial cells *via* downregulation of CXCL17	CXCL17	(45)
miR-299	Downregulated in asthma	TGIF1, ARNT2, FOSB, OAS2	(50)
miR-342	Suppressed inflammation response in human macrophages THP-1 cells		(123, 124)
	Regulates Treg function. Targets NFκβ	NFκβ	(108)
miR-375	Upregulation of TSLP in human bronchial epithelial cells	SOCS	(56)
	Blocked expression of TLR7 in asthmatic patients		(125)
	Upregulated in bronchial epithelial cells in pollutants-induced exacerbations of asthma		(56)
miR-379	Induced by IL-13, regulated cell surface receptor linked signal transduction	ROR1, YBX1, CXCL11	(106)
miR-409-3p	Sex-specific association with FEV_1_/FVC in asthmatic boys	YBX1 HEY2, AhR, CCL28, TLR5	(36)
miR-485	Upregulated in asthma; modulated the TGF-β/SMAD3 Signaling Pathway	TGF-β1, SMAD3	(126)
miR-489	Upregulated in mice model of allergic rhinitis	Not defined	(122)
miR-498	Correlates with IFNγ in asthmatics	IFNγ	(89)
miR-570-3p	Upregulation of CCL4, CCL5, TNFα, and IL-6	CCL4, CCL5, TNFα, IL-6	(37)
miR-628	Dowregulated in rhinosinusitis	Not defined	(127)
miR-643	Regulates expression of IL-17	IL-17, RORA, RORB	(128)
miR-660-5p	Sex-specific association with FEV_1_/FVC in asthmatic boys	SCL46A3, ZNF273	(36)
miR-942	Sex-specific association with FEV_1_/FVC in asthmatic boys	Not defined	(36)
miR-1180	Activates NFκβ	NFκβ	(129)
miR-1248	positive regulator to increase IL-5 expression	IL-5	(49)
miR-1248	Negatively correlates with lung function in asthma	Not defined	(49)
miR-1290	associated with asthma and atopy during pregnancy, interacts with TGF-β signaling	TGF-β1	(130)
	Sex-specific association with FEV_1_/FVC in female asthma patients	NAPSA	(36)
miR-1303	Regulates gene ADAM33, which is related to bronchial hyperreactivity	ADAM33	(59)
miR-3935	suppression of the PGE2-PTGER3 axis	PTGER3	(98)

Since asthma is a disease that occurs at a young age, some studies have focused on circulating miRs correlated to lung function parameters: such as miR-15b, -126, -139, -142, -186, -191, -342, -409, -660, -942, -1290 (36). The identification of these miRs, their sex-dependent association with lung function parameters as well as their interactions could be useful in finding a treatment option for type 2-related diseases. A functional associaten between miR expression and the pathogenesis of asthma has been reported for miR-1248, miR-1291, miR-570-3p, and the previously mentioned miR let-7a, which were specifically expressed in affected lungs of asthmatic patients when compared to healthy subjects (37). The let-7 family is known as a modulator in Th2 inflammation and is believed to have a pro-inflammatory effect as it is abundantly expressed in the airway tissues of asthmatic patients and is involved in regulating the expression of IL-33 (38, 39). In a mouse model, miR-21 was found to be increased and to regulate IL-12, which is known to propagate Th1 polarization (28). By analyzing the serum levels of miR-21 and IL-4 in asthmatic patients and healthy controls, a strong positive correlation was detected, as both were significantly higher expressed in asthmatic patients (40). miR-21 is expressed at high levels in different cell types and can be increasingly be induced *via* STAT3 and NF-κB (41).

miR-19a contributes to the production of Th2 cytokines and was found to be upregulated in airway infiltrating T cells *in vitro* differentiation experiments. It interacts with mRNAs encoding PTEN, SOCS1, and A20 (TNFAIP3) by deregulating and derepressing their signalling pathways (42). Another mechanism involved in maintaining Th1/Th2 balance is the impact of miR-155 on macrophages. Its targets have been shown to include IL-13, SOCS1, and SHIP1. When miR-155 is reduced in macrophages, STAT6 becomes more active, resulting in an alternative M2 phenotype (43). In a miR-155 knockout mouse model, mice tended to develop asthmatic inflammation and increased levels of IL-4 and IL-5 in T cells (44). In addition, an association between miR-3935 and its predicted target gene, the prostaglandin E3 receptor, was revealed, which could mediate allergen-specific immunotherapy (AIT) effects through suppression of the PGE_2_-PTGER3 axis (8). Furthermore, miR-221 was found to be reduced in epithelia and sputum of asthma patients and associated with eosinophilic airway inflammation. Therefore, miR-221 might serve a potential biomarker in allergic inflammation. In house dust mite-challenged mice, airway overexpression of miR-221 suppressed CXCL17 expression and thereby enhanced expression of CCL24, CCL26 and POSTN (45). This could potentially affect the epithelial cytokine-mediated priming of epithelial cells towards an E1 or E2 epithelial response, a phenomenon named after the causative Th1 and Th2-derived cytokines (1, 3, 4, 46).

## Non-coding RNAs of airway epithelial cells in type-2 diseases

A major factor in the pathogenesis of asthma are changes in gene expression and secreted protein patterns in the airway epithelium (1, 46). Several studies have examined the expression pattern of miRs in airway epithelial cells of type 2-related diseases targeting genes such as TNF-α, IL-8, or IL-6 (34). MiR-19, a well-studied microRNA is able to promote type 2 cytokine production of IL-33, IL-5, and IL-13 through direct targeting of the signaling inhibitors PTEN, SOCS1, and TNFAIP3 (A20) (47). These signaling inhibitors are not only regulators of Th2 cells but also of ILC2 cells (47). Further, miR-449 is able to repress the expression of IL-13 in asthma (48). Interestingly, also some miRs are known to correlate with changes in lung function parameters in a sex-specific manner, such as miR-485, miR-660-5p, or miR-942 (36). miR-1248, which can increase the expression of IL-5, was also described to negatively correlate with lung function parameters in asthma (49). However, also miRs like miR-34 (38) or miR-299 (50) downregulated in asthma are known. Downregulation of miR-133a contributed to upregulation of RhoA in bronchial smooth muscle cells of asthmatic patients (51) and alleviates airway remodeling in asthma by targeting IGF1R (52). miR-192 expression was reduced in the peripheral blood of asthmatic patients undergoing allergen inhalation challenge (53). It is further able to attenuated airway remodeling and autophagy in asthma by targeting MMP-16 and ATG7 (54). miR-21 has been shown to be upregulated in allergic airway inflammation and to regulate IL-12p35 expression, which appears to promote Th2 and attenuates Th1 responses by targeting IL-12 expression (28). An important regulator of Th17 responses was identified with miR-182, which was shown to be upregulated upon Th17 differentiation, targeting FoxO family members 1 and 3 (55). miR-375 is associated with an increase in the expression of TSLP in primary bronchial epithelial cells, a key cytokine in asthmatic airway inflammation (56). Moreover, miR-221 was described to correlate with airway eosinophilia in asthma and further increased CCL-24, CCL-26, and POSTN in asthmatic airways (45). Counterregulatory mechanisms could be induced by upregulation of miR-146a, which could potentially enhance the T regulatory (Treg) cell-mediated suppression of Th1 responses and result in unhindered Th2 activation (57). The let-7 family members appear to target IL-13 expression, as downregulation of let-7 could enhance Th2 responses by upregulating IL-13 expression (39, 58). However, also regulators of epithelial genes are known. A prominent example is miR-1303, which regulates the epithelial-derived ADAM33 known to be involved in asthma and related bronchial hyperreactivity (59). Taken together, miRNAs are involved in a number of regulatory processes in airway epithelial cells during inflammation.

## Non-coding RNAs in airway remodeling processes

In addition to airway inflammation, airway remodeling is also a pathological hallmark in asthma, driven by eosinophils, neutrophils, and other inflammatory cells (60–62). Increasing evidence suggests that airway remodeling can occur early in childhood, concomitantly with, but not necessarily subsequent to, airway inflammation. Features of airway remodeling include increased smooth muscle cell mass, thickening of airway walls, and epithelial barrier dysfunction (63). In the remodeling process, matrix metalloproteinases (MMPs) were implicated in the degradation of the extracellular matrix in the process of tissue remodeling. MMP-2 and MMP-9 activities were increased around inflamed airways, which were the main site of tissue remodeling (64). In addition, miR-192-5p can alleviate airway inflammation and airway remodeling in asthma by targeting MMP-16 (54). Furthermore, it has also been reported that airway inflammation and airway remodeling are exacerbated by activation of JNK1/2-MMP-9 pathway associated with *ORMDL3* knockdown in asthmatic mice, suggesting that miR-192-5p mediates airway remodeling and autophagy by other signaling pathways (65). Abnormal expression has been reported for miR-451a in pulmonary diseases associated with remodeling processes, as elevated levels of expression have been demonstrated in patients with chronic obstructive pulmonary disease (COPD) (66). Several miRs, such as miR-19a, miR-142, and miR-221, have been found to be differentially expressed in asthma and associated with airway remodeling (67–69). miR-142-3p in bronchial biopsies from patients with early- or late-onset severe asthma was consistent with a differential WNT signature, suggesting that miR-142 is involved in regulating the balance between proliferation and differentiation of airway smooth muscle cells in asthma, possibly *via* control of WNT signaling (68). Also involved in the WNT pathway is β-catenin, which has been shown to be elevated in *Aspergillus fumigatus*-associated asthma, as the 3′-UTR of the β-catenin transcript is a genuine miR-3162-3p binding site (70). Endogenous miR-3162-3p could aggravate the severity of allergic asthma caused by miR-3162-3p antagomir by alleviating the reduction in β-catenin expression in asthmatic mice and suppressing airway hyperresponsiveness and airway inflammation (70). Furthermore, miR-221 is hypothesized to play a crucial role in driving the differentiation of the Th17/Treg ratio *via* RORγt/Foxp3 by targeting SOCS1 (69). In addition, mir-221 reduced the Th17 cell function by directly inhibiting RORγt/SOCS1 and promoted the function of Treg cells *via* induction of Foxp3/SOCS1 in asthma (69). Asthma-related airway remodeling was reduced by miR-451a overexpression, which was been shown to target ETS1, while miR-451a downregulation promotes differentiation of CD4^+^ T Cells towards Th2 cells through ETS1 upregulation in childhood asthma (71). Among these phases, the airway remodeling phase is irreversible, and directly related to the prognosis of affected children (61).

Additionally, the extracellular matrix plays an important role in asthma-induced airway remodeling, including type I collagen (COL-I) and fibronectin (FN), since secretion of COL-I and FN results in thickening of the basal membrane and subcutaneous fibrosis of the airway, eventually leading to airway remodeling (61). MiR-146a and miR-146b are negative regulators of inflammatory gene expression in lung fibroblasts, epithelial cells, monocytes, and endothelial cells. They negatively regulate the expression of cyclooxygenase-2 (COX-2) and IL-1β (72). These findings suggest that miR-146a and/or miR-146b are able to modulate the expression of inflammatory mediators in airway smooth muscle cells, thereby contributing to the pathogenesis of asthma (72). Elevated levels of miR-378 in the serum of asthmatic children have been shown to promote cell proliferation and resistance to apoptosis. Upregulation of miR-378 promotes smooth muscle cell proliferation and resistance to apoptosis, and increases the expression of COL-I and FN proteins (61).

Thymic stromal lymphopoietin (TSLP) acts as a key epithelial-derived cytokine involved in remodeling of the asthmatic airway. Decreased TSLP expression reduced the production of inflammatory cytokines and thereby inhibited STAT3 expression and phosphorylation. STAT3 upregulation and activation in the airways are closely associated with the onset and development of asthma, while STAT3 activation facilitates TSLP in asthma-associated airway remodeling (73). TSLP can be negatively regulated by miR-19b by binding to the upstream non-coding region of TSLP (74). Therefore, miR-19b is able to reduce airway remodeling, airway inflammation, and levels of oxidative stress by inhibiting TSLP signaling through STAT3. Since TSLP as well as the other epithelium-derived alarmins IL-25 and IL-33 also affect the expression and secretion of IL-5 and IL-13 from innate lymphoid cells type-2 (ILC2), it directly influences the miR-mediated regulation of immune cells.

## Non-coding RNAs of immune cells in type-2 diseases

The function of ILC2s by targeting TSLP can be regulated by miR-375, which has been reported to be downregulated in patients with Th2-associated diseases such as atopic dermatitis, ulcerative colitis and allergic rhinitis (75). Systemic miR-15b levels were shown to be associated with lung function, while miR-16-5p levels correlated with bronchial hyperresponsiveness (BHR). Local T cells from asthma patients expressed diminished levels of miR-19a-3p, the upregulated levels of which promote the production of Th2 cytokines (42). miR-126 increases GATA-3 expression in T cells in an indirect manner that might promote a Th2 response and is believed to increase IL-13 levels. This suggests that it is associated with an excessive activation of Th2 cells in asthmatic children (76). In contrast, miR-21-3p detected in exhaled breath condensate of asthmatics showed reduced levels compared to healthy controls (77). In addition, a pediatric study observed elevated levels of miR-191-5p targeting CEBPB, which correlated with the lung function parameter FEV1/FVC (Tiffeneau) (36). Furthermore, miR-223-3p was elevated in asthmatics compared to healthy controls and correlated with lung function parameters (36). MiR-210-3p has been shown to be involved in mast cell activation (78), while miR-223-3p is involved in neutrophil maturation and function by inhibiting IL-18 expression in macrophages, suggesting a fine-tuned mechanism involving inflammatory IL-18-induced neutrophil extracellular traps (NETs) (79).

Since both the innate and adaptive immune responses show altered IL-13 levels, the expression of the interlinked microRNA could also be altered. IL-13, produced by Th2 cells but also by group 2 ILC2s, has been found to increase neutrophil-derived PGE2 levels by upregulating COX-2 gene expression (8). The suppressive effects of AIT on type-2 immune responses not only affect Th2 cells but also induce a shift from ILC2 to ILC1. For example, miR-155 can affect the Th1/Th2 balance by targeting IL-13RA, SOCS1, and SHIP1 of macrophages, since specific modulation of miR-155 expression may be able to reduce exaggerated inflammation (80). In addition, IL-10 induces miR-187, which is able to negatively regulate the expression of the pro-inflammatory mediators TNF-α, IL-6 and IL-12p40, as it directly affects the stability and translation of TNF-α mRNA targets and indirectly decreases IL-6 and IL-12p40 expression *via* down-modulation of IκBζ, a major regulator of the transcription of these two cytokines (81). Furthermore, miR-21 expression increased remarkably in an OVA-induced asthma model and suppressed the expression of IL-12/STAT4 proteins (82). Therefore, miR-21 expression increased significantly and both IL-12p70 and IL-12p35 were down-regulated in mice with OVA-induced asthma (82). In addition, suppression of miR-21 by intranasal administration of the antagomir improved asthma symptoms including airway inflammation and BHR, inhibited Th2 polarization of CD4^+^/CD8^−^ cells, and altered cytokine levels in BAL fluid (83). These immune cell-related regulatory changes are key targets of treatment options such as AIT, which also initiate changes in miR expression and regulation.

## Non-coding RNAs in allergen-specific immunotherapy for inhalant allergens

miRs have been intensively studied in recent years in the context of allergic and respiratory diseases (9, 84). There is a wealth of research focusing on the role of miRs in the pathogenesis of asthma and allergies, and thus to our understanding and delineation of disease endotypes (85) in preclinical (28) and clinical (86) settings.

**Figure 1:**
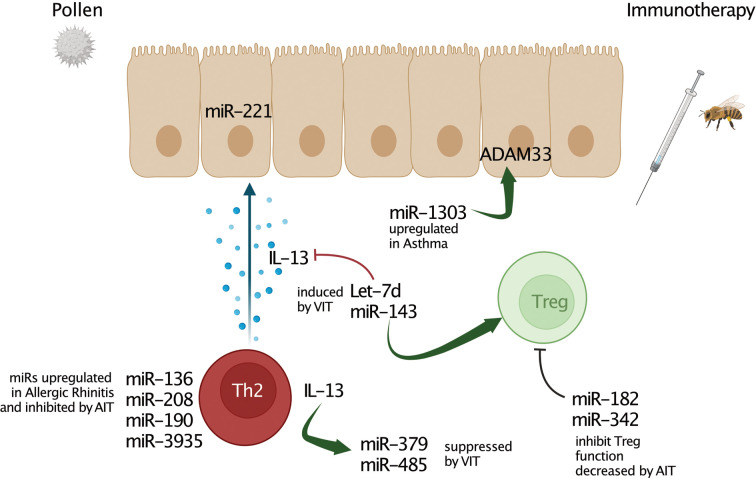
MicroRNA interactions in allergic inflammation and allergen-specific immunotherapy (AIT). Several miRs play a crucial role in regulating multiple processes and characteristics of allergy-associated disease pathology. Deregulation of multiples miRs affects to inflammatory processes, Th1/Th2 response balance, cytokine, chemokine, lipid mediator production, and remodelling processes. However, miRs up-regulated by immunotherapy such as Let-7d or miR-143 can also alleviate airway inflammation and decrease pro-allergic cytokine production by promoting regulatory T cells (Tregs). These miRs directly inhibit type 2 key mediators such as IL-13. Further, negative regulatory association of miR-3935 and its predicted target gene, the PGE_2_ receptor EP3, revealed this miR as a potential AIT-mediated mechanism in the airways of AA patients. Moreover, there is also evidence for miRs targeting regulatory factors of asthma such as ADAM33.

No less important was the work aimed at elucidating the differences in specific miR levels in health and disease (40), revealing the association between specific miRs and disease risk, severity (71, 87) and exacerbation (88). Further, also characterizing of miR profiles of patients presenting with one or two allergic comorbidities (89), and lastly predicting the clinical remission of childhood disease in early adulthood (71). Further, the effect of prophylactic sublingual immunotherapy revealed changes of miRs in asymptomatic subjects, who were sensitized to cedar pollen (90). As cedar pollinosis is the predominant seasonal allergic rhinitis in Japan, it is of interest that miR-223 was significantly up-regulated in pollen season and let-7b was down-regulated in sensitized subjects and may possibly mirror the exposure to antigen during pollen season (90). Another important line of research lies in the investigation of miRs as potential biomarkers for monitoring allergy treatments and therapeutic outcomes, particularly in relation to resource-intensive therapies, such as AIT (Figure 1; Table 2).

**Table 2:** Micro-RNAs involved in pathogenic mechanisms of allergen immunotherapy.

miRNA	Potential function in pathomechanism	Regulation by AIT	References
let7d	Decreased expression of TLR-4, IL-6, and IL-13	Up	(104)
miR-18a	Decreased levels in asthma	Unchanged	(104)
miR-23a	Associated with tolerogenic dendritic cell activity and Treg responses	Up	(104)
miR-29c	Differentiation of T cells, regulation of cell proliferation and apoptosis	Up	(104)
miR-34b	Down-regulated in asthma	Up	(104)
miR-143	Stimulation of FoxP3, stimulates proliferation of CD25+ CD4+ lymphocytes, downregulated IL-13	Up	(104, 116)
miR-182	Differentiation of Th17 cells	Down	(55, 104)
miR-190	Upregulated in allergic rhinitis	Down	(97)
miR-204	Regulates bronchial smooth muscles cells proliferation	Up	(100, 105)
miR-208	Upregulated in allergic rhinitis	Down	(97)
miR-299	Downregulated in asthma,	Up	(50, 100)
miR-342	Regulates Treg function, targets NFκβ	Down	(100, 108)
miR-375	Upregulation of TSLP in human bronchial epithelial cells, decreased by VIT	Down	(56, 104)
miR-379	Induced by IL-13, regulated cell surface receptor linked signal transduction	Up	(100, 106)
miR-485	Upregulated in asthma; modulated the TGF-β/SMAD3 Signaling Pathway	Down	(100, 126)
miR-489	Upregulated in mice model of allergic rhinitis	Down	(100, 122)
miR-601	Upregulated in allergic rhinitis	Unchanged	(104)
miR-628	Controls TLR signaling	Up	(100, 131)
miR-643	Regulates expression of IL-17	Down	(104, 128)
miR-1201	Upregulated in allergic rhinitis	Unchanged	(104)
miR-1303	Regulates gene ADAM33, which is related to bronchial hyperreactivity	Up	(59, 104)
miR-136	Upregulated in allergic rhinitis	Down	(97)
miR-3176	Downregulated in asthma	Up	(98, 132)
miR-3935	Mediated AIT effects through suppression of the PGE2-PTGER3 axis	Up	(98)
miR-4664-3p	Linked with HIF1A	Up	(98, 133)
miR-6824-3	Associated with TLR pathway genes	Up	(98, 134)

Allergen-specific immunotherapy is an effective disease-modifying therapy used for the treatment of allergic sensitizations, however of limited use in severe or uncontrolled asthma. Targeting specialized treatment regimens, individual genetics, environment, and lifestyle must be incorporated in therapeutic decision-making (91). These include comprehensive and accurate allergy diagnostic testing tools, a better understanding of the mechanisms, the identification of biomarkers to predict and monitor response, and the development of safe, effective, affordable and convenient treatments (91). In addition to changes in decreased allergen-specific IgE and increased allergen-specific IgG production, AIT has been associated with several other immunological events, including alterations in allergen-specific T- and B-cell population and associated cytokine responses, production of antibodies capable of blocking allergen presentation, thought to be of the IgG_4_ subset, reduction in tissue eosinophils and mast cells, as well as decreased basophil activation (91–93). Some published works observed these immunological events take place at different time points in the AIT course (93, 94). As mast cell and basophil desensitization are early events in AIT treatment, they are followed by the induction of IL-10 producing T and B regulatory cells that induce a B cell isotype switch that shifts the immunoglobulin production from IgE to IgG. There is also a shift in cytokine production which results in suppression of effector type 1 and type 2 cells reflecting the antagonistic cytokine pattern (1, 93, 95). These changes in the cytokine milieu are in turn affecting also e.g. the airway epithelium, which was shown to express an antagonistic response following a cytokine-dependent polarization (1, 2, 93). AIT has been used in clinical practice for more than 100 years, and although it has been used very successfully to treat severe insect sting reactions with a response rate of approximately 90%, it has variable responses in patients with respiratory allergies and is considered moderately effective (96). However, considering this fact, it must be taken into account that the efficacy of therapy for insect venom allergy and allergies to inhaled allergens are assessed in different ways. Insect venom allergy is a disease with infrequent or even absent allergen exposure for years and the efficacy of VIT is assessed based on the lack of systemic sting reaction. In contrast, allergies to inhalant allergens are characterized by seasonal or perennial allergen exposure, and treatment efficacy is assessed based on a symptom and/or medication score. Due to these differences, the efficacy of VIT and AIT with inhalant allergens is difficult to compare in a purposeful manner. With the advent and rapid development of miR technologies, the question of AIT efficacy in airway diseases and its biomarkers thas been revisited in several studies. Specjalski et al. determined the expression of 48 miRs in whole blood samples from 16 allergic rhinitis (AR) patients receiving grass pollen AIT and seven healthy controls to establish the possible correlation between miR upregulation and clinical outcomes (97). AR patients showed upregulation of miR-136, miR-208, and miR-190 compared to healthy controls, but no differences between good and poor responders were observed after six months of treatment, despite the overall reduction in pro-inflammatory miRs.

Recently, Jakwerth et al. sought to decipher the effects of grass pollen AIT on miR expression in the sputum of AR patients with or without asthma (98). While over two thousand miRs were upregulated in patients compared to controls, this number was only four in AIT-treated individuals. The prostaglandin EP_3_ receptor, which is the target of one of those upregulated miRs, was downregulated in AIT-treated compared to untreated patients. Of note, PGE_2_ levels were elevated even in the AR group, decreased after AIT, and correlated with type 2 markers in sputum and symptom severity.

Although miR-based approaches have enabled considerable progress in improving our understanding of the role of finely tuned mechanisms regulating gene expression in health and in a variety of allergic diseases, there remains an unmet need for large-cohort double-blind placebo-controlled AIT studies to determine which miR signatures are the most promising candidates for identifying therapy responders and predicting treatment outcomes in the clinical practice.

## Non-coding RNAs in venom-specific immunotherapy

Like AIT with inhalant allergens, venom-specific immunotherapy (VIT) is a highly effective causative treatment for allergy that can restore allergen tolerance and protect the patients against future potentially fatal allergic sting (99). In contrast to allergy e.g. to airborne allergen sources, venom allergy is not considered an atopic disease. To date, very few studies addressed changes in the expression of miRs over the course of VIT (Table 2).

A 2016 study examined the changes in 740 miRs 24 h after completion of the up-dosing phase of VIT using an ultra-rush protocol in seven patients with yellow jacket venom allergy (100). Out of the 740 miRs, 440 could be detected in the peripheral blood of the patients. Five miRs had significantly altered expression 24 h after completion of the up-dosing phase compared to baseline. A significant increase was observed for miR-299 and miR-29c and significant decreases for miR-370, miR-539, and miR-502-3p, none of which were previously mentioned in the context of allergen-specific immunotherapy. Interestingly, the miR-29 family is expressed in T and B cells and thus might be involved in a variety of immunological responses (101, 102), while miR-502-3p is induced by IL-4 (103). In addition, the same study found another 62 miRs that changed 2-fold in some patients, although these changes were not significant. Nevertheless, these miRs could be interesting candidates in connection with the protective effect of VIT, since they are associated with e.g. stimulation of FoxP3 expression and CD4 ^+ ^CD25^+^ lymphocyte proliferation (up-regulation of miR-143) or inhibition of IL-13 and IL-13Rα1 expression (up-regulation of let-7d and miR-143) (58, 73).

In a follow-up study, the same authors addressed changes in miR expression after three months of the maintenance phase in 5 patients with yellow jacket venom allergy (104). At this time point, six miRs showed significantly lower and 11 higher expressions compared to the baseline before the start of VIT. Again, it was found that miR-143 and let-7d are up-regulated by VIT. Additionally, among the most upregulated miRs was miR-34b, which has been shown to be downregulated in asthma (38) and suppressed by IL-13 (48). Other upregulated miRs included amongst others miR-1303, which regulates ADAM33 (59), miR-204, which regulates bronchial smooth muscle cell proliferation (105), miR-299, which is down-regulated in asthma (49), IL-13-induced miR-379 (106) and miR-485 up-regulated in asthma (102). Significantly down-regulated miRs included miR-182, which promotes clonal expansion of activated Th cells and regulates Treg function (12, 107), miR-342, which regulates Treg function (108), and miR-375 up-regulated in bronchial epithelial cells during pollutant-induced asthma exacerbations (56).

Limitations of the studies described above in elucidating potential miR-associated mechanisms contributing to the high therapeutic efficacy of VIT and long-lasting allergen tolerance include the small patient numbers and early sampling time points. Karpinski et al. compared the expression pattern of 2549 miRs from 13 patients with venom allergy one year after the start of VIT with the baseline values before VIT (109). Here, both correlation and principal component analysis indicated a limited effect of VIT on the overall miR expression pattern. After 12 months of VIT, the expression pattern in whole blood was broadly similar to that observed before VIT. Taking into account the differences between the studies, the authors conclude that the short- and long-term effects of VIT on the miR pattern appear to differ. Therefore, larger controlled studies are needed to examine the contribution of miRs to the tolerogenic effects of VIT in both immediate and long-lasting effects.

## Conclusion

New techniques have led to major advances in the field of miR research, resulting in a deluge of information and long lists of potentially interesting miRs. However, mechanistic studies on miR targeting and function are often lacking. Furthermore, miRs alone may not or only rarely be the key to explaining the pathology of asthma or allergic diseases, so that complex interaction networks are required to elucidate the pathogenesis of diseases and their heterogeneity. The expression of miR in certain cell populations, in defined disease states, in therapy models and in certain phenotypes must also be researched intensively to decode the pathophysiological consequences of altered miR expression, for example in allergic diseases. However, this review article summarizes the critical role of miR in the pathogenesis of asthma and allergic diseases and their associated comorbidities, making them interesting targets for therapeutic interventions such as allergen-specific immunotherapy. Since asthma and allergies are very heterogeneous diseases and current treatments are still ineffective in controlling severe forms of these diseases, there is a great need for precise and more effective therapies based on a deep understanding of the disease-underlying mechanisms. Interest in using miR profiles as biomarkers in lung diseases continues to grow. However, in order to implement the use of miRs as biomarkers, they should be very specific for certain signaling pathways or sub-cell types that, through their association with the disease or therapy underlying mechanisms, allow for a more detailed characterization of particular disease phenotypes. The position that the individual miRs can have in the hierarchy as biomarkers for predicting the course of a disease or the response to therapeutic interventions must be clarified in the coming years.
